# The natural progression of a fistulizing gallstone resulting in massive gastrointestinal hemorrhage and Bouveret syndrome, a rare case

**DOI:** 10.1007/s12328-019-01054-x

**Published:** 2019-10-25

**Authors:** Elmer Hoekstra, Maarten Willem van den Berg, Roeland Andreas Veenendaal, Rogier Stuyt

**Affiliations:** 1 Leids Universitair Medisch Centrum (LUMC), Albinusdreef 2, 2333 ZA Leiden, The Netherlands; 2 Haga Ziekenhuis, Els Borst-Eilersplein 275, 2545 AA The Hague, The Netherlands

**Keywords:** Gallstones, Bouveret syndrome, Cholecysto-duodenal fistula

## Abstract

Gallstones are seen very common, especially in the Western World. While most patients are asymptomatic, gallstones can cause life-threatening complications. Here, we present a rare and nearly fatal complication of gallstones, showing the natural progression of gallstone disease. With two very unusual complications of gallstones which occurred in the same patient. Massive gastrointestinal bleeding, and the Bouveret syndrome.

## Introduction

In the Western world up to 15% of the adults have gallstones. While most of them remain asymptomatic, gallstones can result in several complaints with the biliary colic as the most prevalent. Other complications such as symptomatic cholecystolithiasis, cholecystitis, cholangitis, and biliary pancreatitis is a also quite common problem. More rare complications include gallstones fistuling towards the duodenum, with the possibility of causing a gastrointestinal obstruction. Here we present a rare and nearly fatal complication of gallstones, showing the natural progression of gallstone disease.

## Case report

A 71 year-old male, with a previous history of severe obesity (Body Mass Index 45 kg/m^2^), hypertension, and a moderate performance status, presented to the emergency department with rectal blood loss and symptoms of a hypovolemic shock. Besides amlodipine for his hypertension, the patient did not use any medication, especially no anticoagulants. In the weeks before presentation he had loss of appetite, complaints of heartburn, and epigastric pain. Just before presentation he had large amounts of rectal blood loss and collapse. On physical examination he was pale and clammy with cold peripheral extremities, hypotensive (blood pressure 85/40 mmHg), with a tachycardia of 150/min. Digital rectal examination revealed dark red blood. Blood tests showed Hemoglobin 6.6 mmol/L, MCV 89 fL, Thrombocytes 341 × 10^9^/L, Leucocytes 15.0 × 10^9^/L, Urea 18.6 mmol/L, Creatinine 108 µmol/L.

**Fig. 1:**
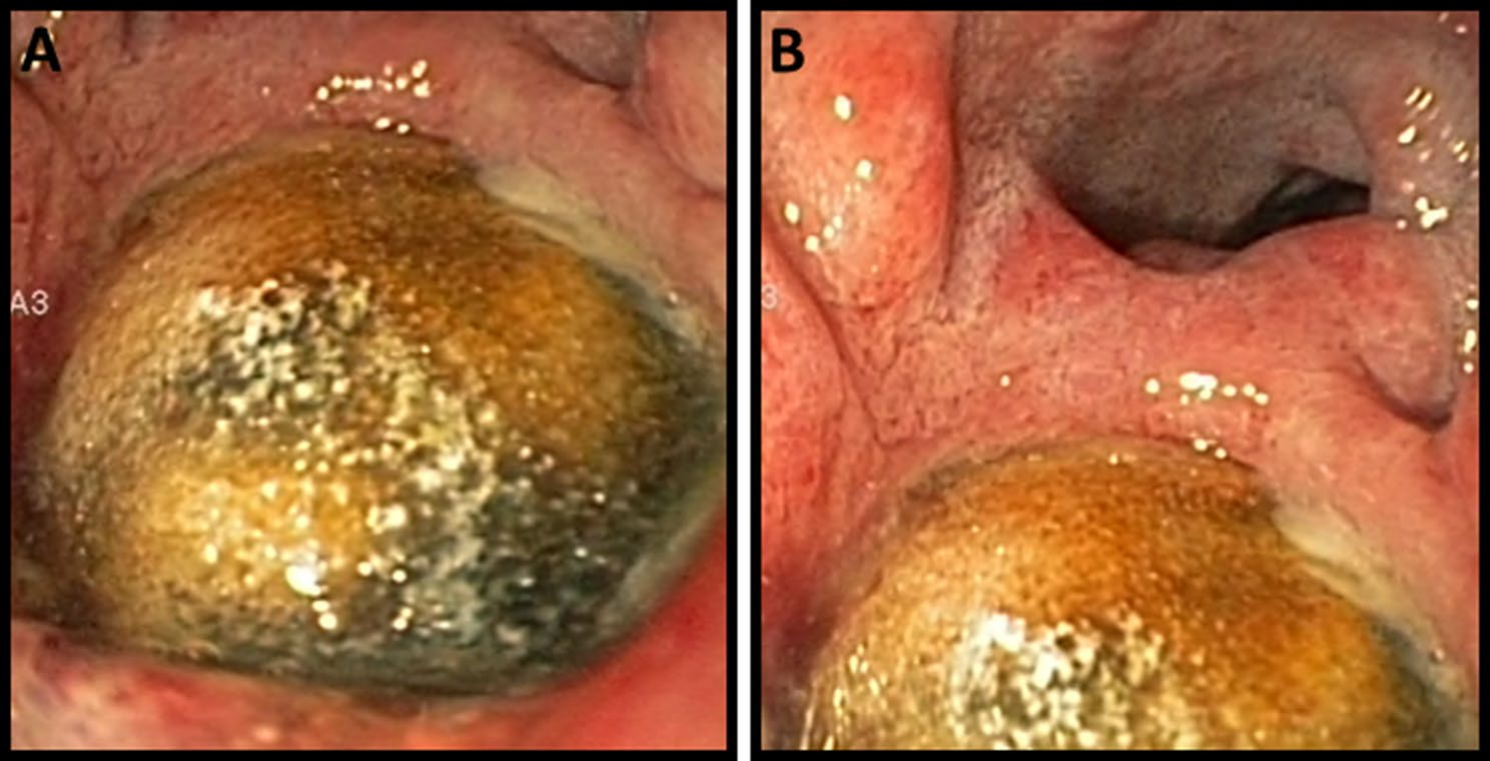
Pictures of esophagogastroduodenoscopy performed under the suspicion of an upper-GI-bleeding. **a** The fistulizing gallstone, stuck in the duodenal bulb, with a vulnerable/ulcerative rim. **b** More overview of the duodenum with the passage towards the second part of the duodenum

**Fig. 2:**
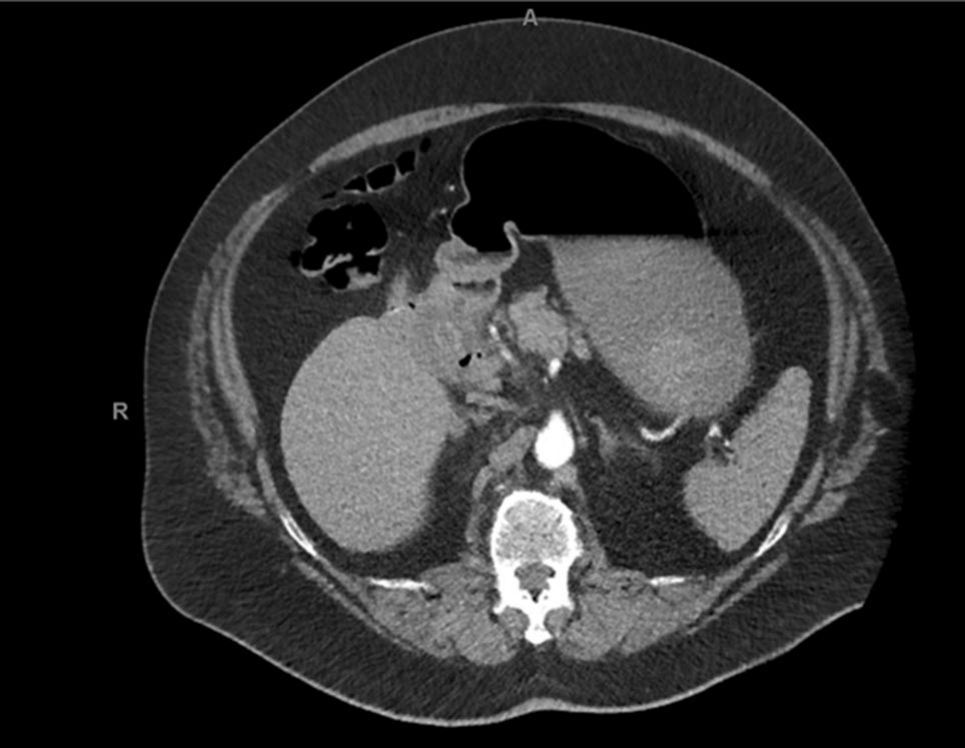
Image of computer-tomography scan with contrast, where no active bleeding was observed. The fistulizing gallstone is visible in the image

Under the suspicion of an acute upper gastrointestinal bleeding, a bolus of intravenous proton pump inhibitor (PPI) followed by continuous infusion was initiated, and patient was admitted to the ICU department for hemodynamic stabilization with fluid resuscitation and vasopressor therapy. Subsequently, an esophagogastroduodenoscopy was performed which showed a cholecysto-duodenal fistula (to the bulbus), with a gallstone of approximately 3 cm in diameter fully obstructing the lumen of the fistula, and normal passage towards the second part of the duodenum (Fig. 1). The fistula rim appeared ulcerative due to the compression of the gallstone, and showed signs of recent bleeding. However, at that time there was no active bleeding or visible vessel suitable for endoscopic treatment. Rectal blood loss subsided spontaneously and we continued conservative treatment with PPI-therapy via continuous intravenous administration, a nil per mouth regime, and blood transfusion with two units of packed cells. The next day patient redeveloped signs of hypovolemic shock, again accompanied by large amounts of melena. After airway intubation, a new esophagogastroduodenoscopy was performed, which showed diffuse blood loss, originating from the pressure ulcers that developed around the fistula rim. This resulted in diffuse blood loss from around/under the gallstone which was stuck in the fistula rim. Again, there was no visible vessel or pseudoaneurysm, targetable for direct endoscopic intervention. Hemostasis was achieved with endoscopic application of hemostatic spray (Hemospray, Cook Medical) as salvage therapy. With the intention of performing radiology guided transcatheter arterial embolization in order to achieve definitive hemostasis we endoscopically placed two hemoclips (Resolution, Boston Scientific) at the adjacent duodenal mucosa for radiological marking. Directly afterwards a computo-tomography was performed, which showed no active bleeding (Fig. 2). In consultation with patient and family, it was decided that surgical intervention was not desirable due to the severe obesity and moderate performance status. A non-escalating wait-and-see policy was agreed, knowing that re-bleeding or gall-stone ileus may occur in the future.

**Fig. 3:**
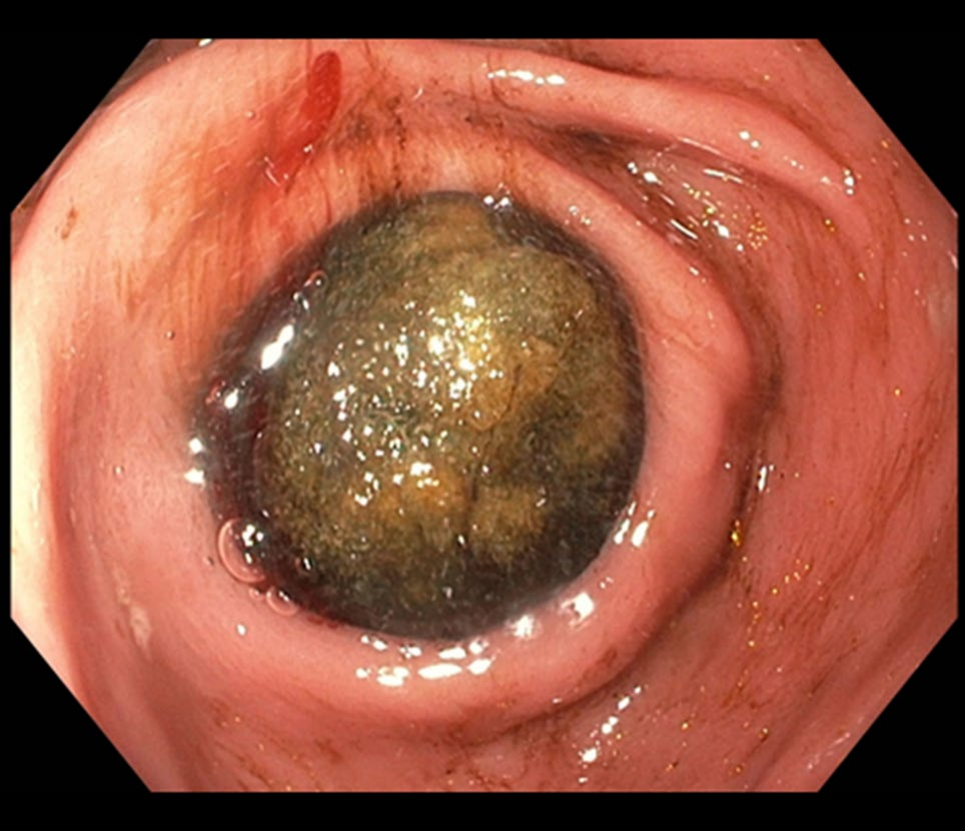
Picture of esophagogastroduodenoscopy performed under the suspicion of a gastric-outlet syndrome. The for-mentioned gallstone is observed just post-pyloric, causing a gastric outlet stenosis; M. Bouveret

**Fig. 4:**
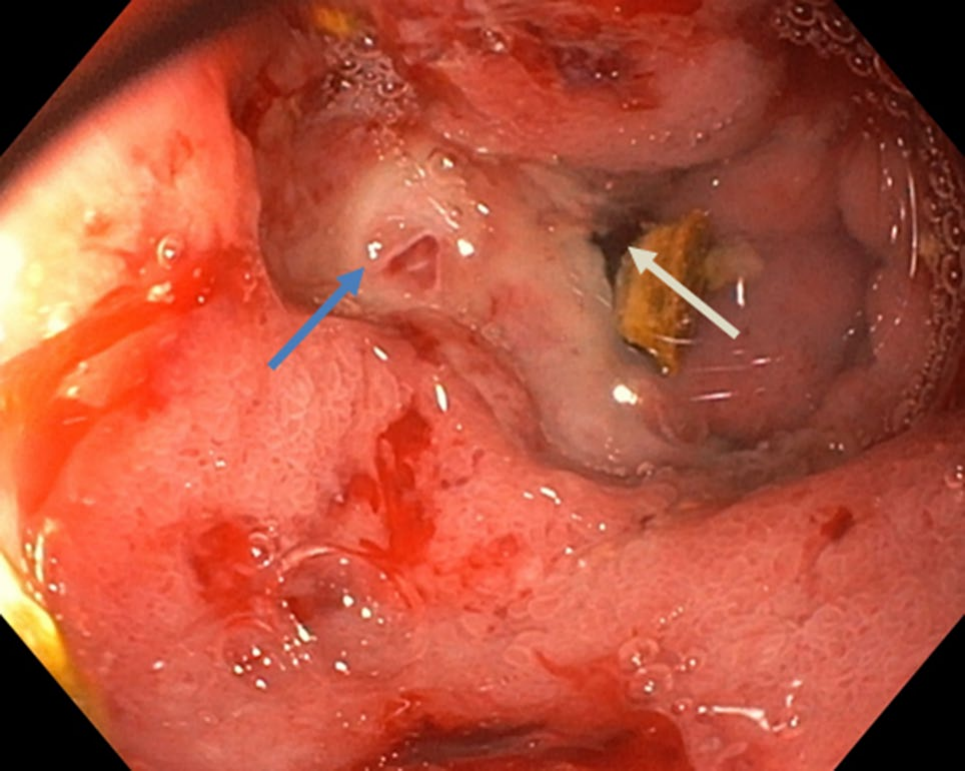
Duodenum after the removal of the gallstone. The blue arrow shows the pressure ulcer formed by the gallstone. The white arrow shows the fistula opening

**Fig. 5:**
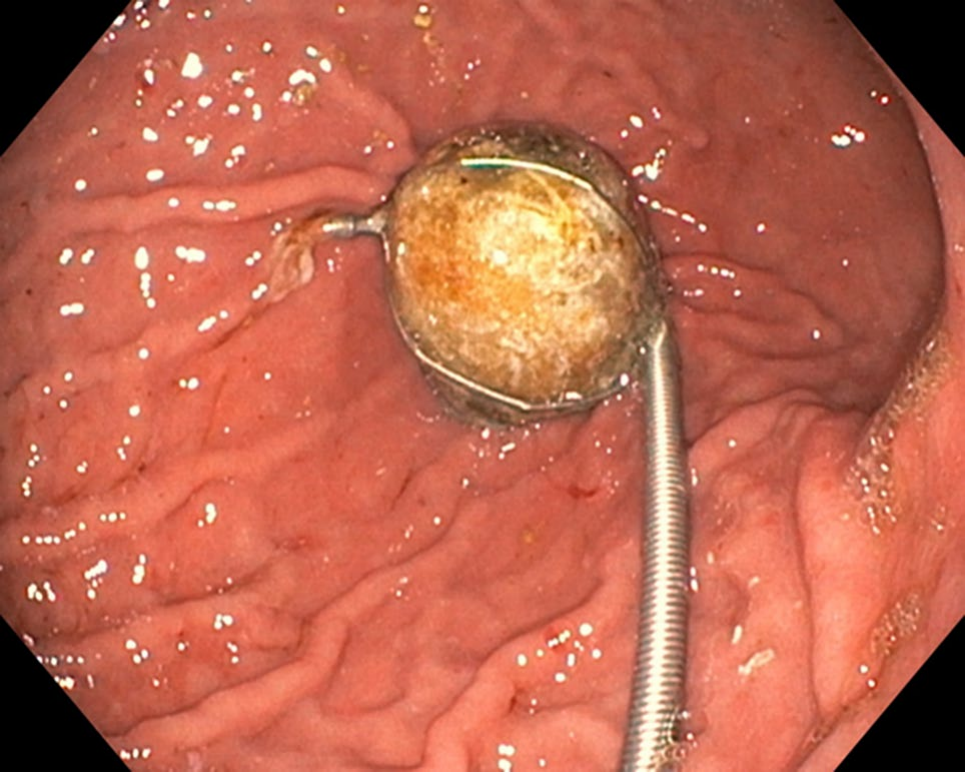
Attempt to crush a large fragment of the gallstone using the Soehendra Crusher basket, however the fragment is too hard to crush

However, after above mentioned treatment, patient recovered surprisingly well and was discharged from the hospital after 7 days with twice daily oral PPI. Two months later patient presented to our outpatient clinic with complaints of nausea and vomiting, without any abdominal pain. Esophagogastroduodenoscopy was performed, showing extensive food stasis in the stomach and esophagus resulting in reflux esophagitis grade D (LA classification). After endoscopic removal of the food debris and fluid retention the previously found gallstone was seen just post-pyloric causing complete obstruction of the duodenal bulb (Fig. 3). Therefore we made the diagnosis of a classic Bouveret syndrome (gallstone ileus). Again, esophagogastroduodenoscopy under general anesthesia on the operating room was performed with the gastrointestinal surgeon present. Using electrohydraulic lithotripsy (Walz Eletronic, P3 EHL probe) the obstructing stone could be split in half, after which one of the halves came back into the stomach in several fragments. The other half of the stone (3–4 cm) was brought back toward the stomach from the duodenal bulb using a crusher basket. Inspection of the duodenal bulb after stone removal revealed a large pressure ulcer with a fistula (Fig. 4) to the gallbladder from which several smaller gallstones were removed with endoscopic flushing. Then, we performed three attempts of mechanical lithotripsy of the large stone fragment with different types of crushing baskets (Olympus, Soehendra), however all failed because the stone fragment was extremely hard (Fig. 5). In a final effort to treat the patient endoscopically, we tried to remove the large stone fragment through the esophagus using an overtube, however this also failed due to the size of the stone. Subsequently the surgeon performed a gastrostomy via small laparotomy incision for complete removal of the stone fragments. After two nights of clinical observation patient could be discharged in good clinical condition without any complications. In the outpatient clinic follow-up, the patient is completely recovered, and does not experience any complaints.

In conclusion, we here present a rare case showing the natural progression of a large gallbladder stone. First, a fistulizing gallstone stuck in the bulbus duodeni, resulting in a nearly fatal upper gastrointestinal hemorrhage due to vulnerable/ulcerative fistula rim. Second, the spontaneous “birth” of the gallstone from the fistula, resulting in a gastric outlet obstruction syndrome; the classic Morbus Bouveret.

## Discussion

Biliodigestive fistula are a rare complication of gallstone disease, with reported incidence rates of 3–5% in patients with cholelithiasis [1]. Cholecysto-duodenal fistula (CDF) resulting in complications such as hemorrhage or ileus are even more uncommon. Gastrointestinal hemorrhage has been reported only in case reports, with a recent overview of the literature showing only ten cases of upper-GI-bleeding due to CDF [2]. Gastrointestinal obstruction is also rare, since most of the gallstones can pass through the GI-tract spontaneously. Only a small number of the gallstones will cause an ileus by obstruction, where the ileocecal valve is the most common site of obstruction (90%). Only 3% off the gallstone ileus result in gastic outlet obstruction by impacting the duodenual bulb, also known as Morbus Bouveret [3, 4]. Our presented case had both these complications, showing the natural progression of a fistulizing gallstone. The initial treatment was focused on stopping the massive hemorrhage. The definite treatment at that point would be major surgical intervention, with high morbidity and mortality rates, especially in this high-risk patient. Therefore a conservative wait-and-see policy was chosen, knowing that gastrointestinal obstruction could occur. The treatment options for the obstruction include endoscopic and surgical interventions, where endoscopic removal is the preferred option of choice, unfortunately with a rare success rate. Several techniques have been used, such as intracorporeal endoscopic electrohydraulic lithotripsy and crushing baskets [5]. In our patient we were able to split the stone in half using the lithotripsy, but the stone was too hard to crush further with both the lithotripsy, as well as several crusher baskets. Therefore minimally invasive laparotomy was performed to remove the fragments from the stomach.

In conclusion we present the first case of a patient that suffered from two extremely rare but major complications of gallstone disease. Due to his comorbidity we treated this patient with conservative methods. Since the complications of conservative treatment on the hand can be severe, and the post-operative mortality rates on the other hand are substantial, this treatment decision is one that should be reviewed carefully and should be based on individual basis.
